# IMPACT OF LOW PROTEIN DIETS ON GUT HEALTH

**DOI:** 10.1093/geroni/igae098.3666

**Published:** 2024-12-31

**Authors:** Prerana Vaddi, Khristina Young, Molly Easter, Cristal Hill

**Affiliations:** University of Southern California, Leonard Davis School of Gerontology, Los Angeles, California, United States; University of Southern California, Los Angeles, California, United States; University of Southern California, Los Angeles, California, United States; University of Southern California, Los Angeles, California, United States

## Abstract

**Introduction:**

Studies on the gut-brain axis report that changes in the microbiome contribute to age-related diseases. One of the most significant factors affecting microbiome composition is diet. Prior work from our lab has demonstrated that low protein (LP) diets induce fibroblast growth factor 21, improve metabolic health, and extend lifespan in male mice. However, the molecular adaptations of a low-protein diet on gut health and their systemic effects on protecting against age-related diseases remains unclear. Here, we investigated the impact of an LP diet on the microbiome and the associated effects on metabolic and immune health.

**Methods:**

At 15 months of age, female mice (n=12 mice/diet) were fed either a normal protein (NP) or LP diet for 20-weeks. Feces were collected to assess microbiota, species richness, and species diversity. Metabolic profiling on food intake, fasted glucose levels, and glucose tolerance tests were conducted to assess the relationship between microbiome profiles and metabolic health.

**Results:**

Relative abundances of Bacteroides and Defferibacterota were reduced in LP diets and increased in Verrucomicrobiota. Gene set enrichment analyses (GSEA) reveal that mice on the LP diets regulate T-Cell dependent immune and inflammatory pathways. Consistent with our previous studies in male mice, we show that aging female have improved glucose clearance during LP-diet.

**Conclusion:**

Notably, previous studies show Bacteroides species promote T-Cell dependent immune pathways, and Verrucomicrobiota species regulate glucose homeostasis. Collectively, GSEA data, the improvements in glucose homeostasis, and changes in microbiota significantly benefit metabolic and immune health.

